# Protocol for Functional Assessment of Adults and Older Adults after Hospitalization for COVID-19

**DOI:** 10.6061/clinics/2021/e3030

**Published:** 2021-06-07

**Authors:** Caroline Gil de Godoy, Erika Christina Gouveia e Silva, Danielle Brancolini de Oliveira, Amislaine Cristina Gambeta, Elizabeth Mendes da Silva, Camila Machado de Campos, Ana Carolina Basso Schmitt, Celso R. F. Carvalho, Carolina Fu, Clarice Tanaka, Naomi Kondo Nakagawa, Carlos Toufen, Carlos Roberto Ribeiro de Carvalho, Keith Hill, José Eduardo Pompeu

**Affiliations:** I Departamento de Fisioterapia, Fonoaudiologia e Terapia Ocupacional, Faculdade de Medicina FMUSP, Universidade de Sao Paulo, Sao Paulo, SP, BR; II Divisao de Pneumologia, Instituto do Coracao (InCor), Faculdade de Medicina FMUSP, Universidade de Sao Paulo, Sao Paulo, SP, BR; III Monash University, Peninsula Campus, Frankston VIC, Australia

## INTRODUCTION

The novel coronavirus, severe acute respiratory syndrome coronavirus 2 (SARS-CoV-2), which causes coronavirus disease (COVID-19), was first reported in Wuhan, Hubei Province, China, in late December 2019. In January 2020, the World Health Organization declared COVID-19 as a public health emergency of international concern (1). As of March 27, 2021, which was one year after the first case was reported, COVID-19 has already affected 126,726,672 people worldwide and has caused 2,777,337 deaths (2).

COVID-19 affects all age groups and both sexes, although its severity increases in the age groups of more than 50 years, probably because of physiological changes resulting from the aging process and the presence of comorbidities. In addition to older adults, the populations most susceptible to complications are immunosuppressed patients or those with immunological alterations, obesity, diabetes, and hypertension, and those in the recent postoperative period (3).

COVID-19 severity can be classified into five types: asymptomatic, mild, moderate, severe, and critical. Severe cases favor the development of acute respiratory distress syndrome (ARDS). ARDS is caused by the exaggerated release of cytokines in the body, which results in an excessive immune response, thereby interfering with the function of all organs and systems and requiring intensive care in most cases (4).

In the Brazilian population, the survival rate of patients hospitalized for treatment of COVID-19 is 62.05% (5). Approximately 5%-32% of patients with COVID-19 require treatment in the intensive care unit for respiratory and drug support. These interventions added to other treatments such as prone position and prolonged hospitalization, which are necessary in the treatment of COVID-19, can lead to increased immobility time. This causes damage to several structures and functions of the body fundamental to functionality with a negative impact on muscle mass, strength, and function and impairs functional capacity (6-8).

Because COVID-19 is a relatively new disease, only few studies have analyzed the impact of COVID-19 together with the deleterious effects of hospitalization on post-hospital discharge functionality. The scarcity of prognostic studies in patients with COVID-19 shows the need to identify the main effects on the mobility, functionality, lung capacity, and postural control of these patients after hospitalization. For this purpose, it is necessary to develop an evaluation protocol that covers the main factors probably influencing functionality, which allows us to understand the short- and long-term effects of the disease process and hospitalization such that specific prevention and rehabilitation measures can be developed. Therefore, we aim to develop a protocol through a cohort study to assess the functionality of adults and older adults after hospitalization for COVID-19. We believe that hospitalization associated with COVID-19 would have a negative impact on functionality and, although some outcomes would be recovered, some sequelae would remain.

## MATERIALS AND METHODS

### Ethics approval

The study was approved by the Ethics Committee for the Analysis of Research Projects of the HCFMUSP (approval number: 34115720.5.0000.0068).

### Study design and location

This cohort study is in progress, and will evaluate 400 patients who were admitted to referral hospitals for SARS-CoV-2 infection. The study will conduct a 1-year follow-up among these patients at four evaluation time points: 1, 4, 6, and 12 months after hospital discharge.

### Study population

**Figure 1:**
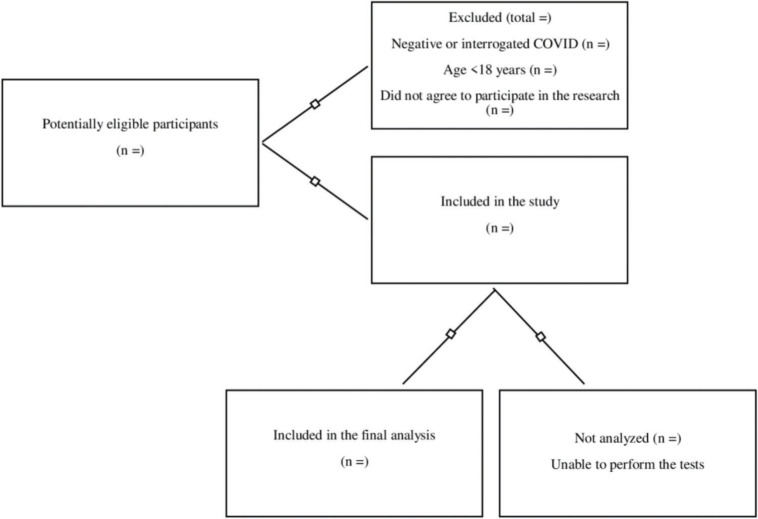
Flowchart of the participant selection process.

Patients will be recruited and interviewed over the telephone regarding their medical records. For participation, the individual should be aged 18 years, agree to participate in this study, and sign the informed consent form. The patient must have been admitted to one of the referral hospitals that are in collaboration with this study, with a confirmed or suspected diagnosis of COVID-19, as well as normal or corrected visual and auditory acuity; the patient must be able to walk, even with the use of an auxiliary device. Those who are unavailable to attend the initial assessment within 30-45 days after hospital discharge or at the proposed collection site or those who do not complete the assessments because of refusal, complications (such as malaise or dizziness), or issues related to not understanding the tests will be excluded from this study (Figure 1).

### Outcomes

The primary endpoint will be the assessment of functionality at the four evaluation points. The secondary outcomes, including those probably indirectly influencing functionality, such as fatigue, anxiety, depression, and cognitive changes, will be measured.

### Data collection, management, and quality control

**Figure 2:**
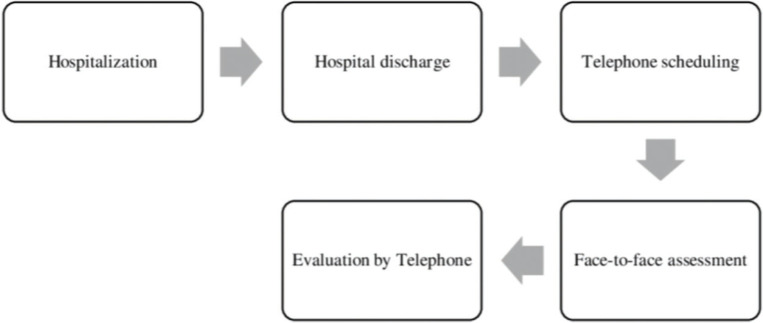
Flowchart of the data collection process.

Figure 2 shows a flowchart of the patient selection process and chronological order of data collection.

The assessment will begin with an interview to collect personal and sociodemographic data (name, age, date of birth, sex, race, marital status, education level, telephone, and address), prior history (Charlson Comorbidity Index and history of falls in the last year), natural history of the disease (day of symptom onset, initial symptoms, history of hospitalization, need for respiratory support and oxygen, prone position, need for emergency room visits, and readmission after hospital release), and whether any skin lesions and sensory changes developed after hospitalization.

**Figure 3:**
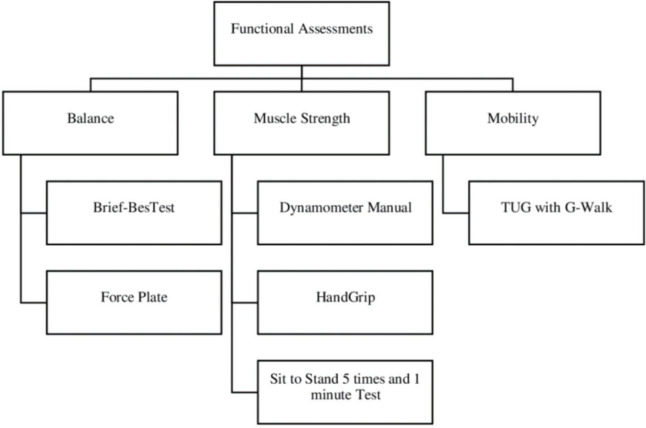
Flowchart of tests applied for functional assessments.

**Figure 4:**
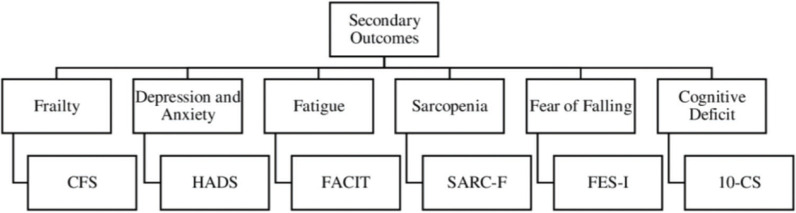
Secondary outcomes that impact functionality.

The functional evaluation (Figure 3) was structured based on the International Functional Classification, which considers function, structure, and body activity as pillars for a complete evaluation. For the choice of questionnaires (Figure 4), we considered the practicality of the application and ease of understanding because except for the 10-point cognitive screener (10-CS), all the questionnaires will be completed through telephone interview. In addition, all questionnaires are validated in Portuguese.

For function and structure, we evaluated global and respiratory muscle strength and resistance. For the evaluation method, we included the Saehan digital manual dynamometer, model DHD-1 (SH1001), for the evaluation of global muscle strength. The test will be performed in both upper limbs for two attempts, and the best measurement will be considered (9,10).

We will perform the 1-min sit-to-stand test to evaluate lower limb resistance. This test counts the number of complete repetitions, that is, standing knee extension and sitting knee at 90° flexion, without using the arms for support while standing up and sitting down, at a speed that the individual feels safe and comfortable. For this test, we will measure peripheral oxygen saturation, heart rate, blood pressure, and Borg for dyspnea and fatigue in the lower limbs before and after the tests. In addition, the time taken to complete five repetitions will be measured to evaluate the muscle strength of the lower limbs (sit and stand test five times) (11,12).

Pulmonary function will be assessed using a portable digital spirometer (Microquark Cosmed, Italy) according to the recommendations of the American Thoracic Society and European Respiratory Society. The absolute and predicted values for the Brazilian population of forced vital capacity (FVC), volume expired in 1s (FEV1), FEV1/FVC ratio, and forced expiratory flow 25%-75% will be analyzed (13,14).

For body activity, we evaluated balance, mobility, and postural control. The balance will be measured in the Behavior Rating Inventory of Executive Function test, which comprises postural reaction, static and dynamic balance, flexibility, and sensory integration tests (15). Each item is scored on a four-point ordinal scale, ranging from 0 to 3 (best score). The final total score is 28 points (16).

Mobility will be evaluated using the standardized timed-up-and-go test as described by Padgett et al. (16). It measures the time taken by a patient to stand up from a chair, walk for 3 m, return, walk again to the chair, and sit down. For accurate execution and more precise data analysis, the G-walk sensor will be used, which is a digital wireless system for gait analysis. It provides data on spatiotemporal parameters (exact total test duration, functional mobility ability, fall risk, and duration of each test phase) and overall kinematics (amount of trunk flexion and extension during the test). The sensor will be placed on a belt at the height of the L5 lumbar vertebra, and through Wi-Fi, the data will be sent to the software for data analysis (17).

In addition, we will evaluate postural control using the portable Horus force platform from the manufacturer Contronic, which has high precision to determine the center of pressure. To perform the tests, the patient's stability limits must first be evaluated. The patient will then be asked to stand for 30s under the following conditions: eyes open and closed on stable ground and eyes open and closed on unstable ground. The software program will then analyze the data of the stability limit area (SL, mm), trust ellipse area (TE, mm2), path length (PL; mm), total average PL speed (mm/s), and TE/SL ratio (%) (18).

Finally, functionality will be assessed using the Barthel Index, which evaluates an individual's performance in activities of daily living. It is considered reliable to monitor changes in functionality patterns and to assess functional independence in daily life tasks. The sum of the points referring to the functions will classify the patients’ degree of dependence (19,20).

Fatigue, sarcopenia, frailty, fear of falling, anxiety, depression, and cognition were included in the protocol to analyze all aspects that could influence functionality. For the evaluations, we will use the following questionnaires:

- **Functional Assessment of Chronic Illness Therapy Fatigue Scale** for fatigue: This questionnaire covers physical, functional, and emotional fatigue and social consequences of fatigue. It contains 13 items, with five responses ranging from “None” to “Very Much.” Items are scored from 0 to 4, added, multiplied by 13, and divided by the number of items effectively answered. The overall score ranges from 0 to 52, with higher scores reflecting less fatigue (21).
- **SARC-F questionnaire** for sarcopenia: This questionnaire comprises five objective questions on activities of daily living and history of falls in the past year. It aims to identify individuals at an increased risk of sarcopenia but not to diagnose sarcopenia (22,23).
- **Clinical Frailty Score** for frailty: This instrument comprises nine clinical items, in which patients can be classified as frail, pre-frail, and non-frail, according to the observation of a healthcare professional and the verification of patient information (24).
- **Falls Efficacy Scale International** (FES-I) for fear of falling: Adapted by the Prevention of Falls Network Europe, the FES-I contains six questions in addition to that in the original scale to assess external activities and social interaction. This questionnaire assesses 16 activities, and for each item, the patient must choose among four options. The points are added up, and the higher the score, the greater the fear of falling (25,26).
- **Hospital Anxiety and Depression Scale** (HADS) for anxiety and depression: This tool is used to identify cases (possible or probable) of mild anxiety and/or depression disorders in non-clinical populations. It comprises 14 items divided into two subscales: HADS-Anxiety and HADS-Depression (27,28).
- **10-point cognitive screener** (10-CS) for cognition: This is a brief screening instrument to detect cognitive impairments. It assesses temporal orientation, category fluency, and memory (29).

### Statistical analyses plan

The results will be presented using descriptive statistics, such as means, standard deviations, 95% confidence intervals, and interquartile ranges. The participants will be divided into groups according to the following criteria: age group, sex, number of comorbidities, and number of medications.

The prognoses will be compared among group using repeated-measures analysis of variance and Bonferroni post-hoc test.

Multiple regression analyses will be performed to study the risk factors for falls and readmission.

All analyses will be performed using SPSS software, adopting a significance level of 5% (*p*<0.05).

Sample size will be calculated to better interpret the clinical significance of the results.

## DISCUSSION

Our study will characterize and follow-up the clinical course of patients after hospitalization for COVID-19 based on their functionality, taking into account not only physical aspects but also other influencing factors such as mood and cognition.

The results of this cohort study will allow better understanding of the impact of hospitalization for COVID-19 on the functionality of the low/middle-income population of a country whose health systems have restricted resources and a reduced number of health professionals.

We will also be able to understand the impact of the pandemic on the outpatient setting and need for investment, both in financial resources and in structure and staff, for the rehabilitation of these patients. We will also be able to observe whether the changes can be reversed because we will observe the patients for 1 year.

Our data will be compared with those available in the current literature on the deleterious effects of hospitalization on adult and elderly patients and on the rehabilitation of patients with post-intensive care syndrome, trying to understand what may be potentiated by the pathophysiology of COVID-19.

Furthermore, our study provides a basis for the functional assessment of patients after hospitalization for COVID-19, allowing the design of clinical trials, raising of relevant research questions, and the development of models to prevent the changes that lead to functional impairments, which can be applied to any new disease outbreaks.

## CONCLUSIONS

We conclude that a functional assessment protocol is important for the development of health policy strategies that will improve the distribution of financial and human resources as well as serve as a source for many research questions that still need to be answered.
